# The effect of insulin-like growth factor 1 on the recovery of facial nerve function in a guinea pig model of facial palsy

**DOI:** 10.1186/s12576-020-00755-0

**Published:** 2020-06-08

**Authors:** Motoyasu Sugiyama, Tsukasa Ito, Takatoshi Furukawa, Atsushi Hirayama, Seiji Kakehata

**Affiliations:** 1 Department of Otolaryngology, Head and Neck Surgery, Yamagata University Faculty of Medicine, 2-2-2 Iida-Nishi, Yamagata-shi, Yamagata 990-9585 Japan; 2 Public Health, Department of Social Medicine, Graduate School of Medicine, Osaka University, 2-15 Yamadaoka, Suita, Osaka 565-0871 Japan

**Keywords:** Peripheral facial nerve palsy, Insulin-like growth factor 1, Electroneurography, Physiological function, Gelatin-based hydrogel

## Abstract

The efficacy of insulin-like growth factor 1 (IGF-1) in the treatment of peripheral facial nerve palsy was investigated using an animal model. The facial nerve within the temporal bone was exposed and compressed by clamping. The animals were treated with either IGF-1 or saline which was topically administered by a gelatin-based sustained-release hydrogel via an intratemporal route. The recovery from facial nerve palsy was evaluated at 8 weeks postoperatively based on eyelid closure, complete recovery rate, electroneurography and number of axons found on the facial nerve. IGF-1 treatment resulted in significant improvement in the changes of the degree of eyelid closure over the total time period and complete recovery rate. A separate study showed that IGF-1 receptor mRNA was expressed in facial nerves up to 14 days after the nerve-clamping procedure. IGF-1 was thus found to be effective in the treatment of peripheral facial nerve palsy when topically applied using a sustained-release gelatin-based hydrogel via an intratemporal route.

## Introduction

The facial nerve (cranial nerve VII) primarily controls the muscles of facial expression, and peripheral facial nerve palsy causes dysfunction of facial movements, typically on one affected side, such as the inability to close the eye, drooling from the corner of the mouth, and facial asymmetry. The most common cause of peripheral facial nerve palsy is Bell’s palsy followed by Ramsay Hunt syndrome. The etiology of Bell’s palsy has been the subject of considerable debate with some characterizing it as idiopathic, while others point to the herpes simplex virus and even others point to the varicella zoster virus (VZV) [1–3]. Bell’s palsy may actually not be caused by a single factor overall, but the cause may differ between patients. In contrast, Ramsay Hunt syndrome is widely acknowledged to be caused by the reactivation of the latent VZV [4]. Latent viral reactivation is generally pinpointed as the cause of swelling of the facial nerve within the narrow confines of the facial canal of the temporal bone which subsequently damages the nerve. Patients with severe peripheral facial nerve palsy due to Bell’s palsy are usually treated with steroids which may be combined with antiviral drugs [5] while patients with Ramsay Hunt syndrome are often treated with steroids and/or antiviral drugs though a standard treatment protocol has yet to be established due to the small number of patients [6, 7]. In severe cases which are not anticipated to respond to drug therapy, facial nerve decompression surgery is sometimes performed [8]. However, some cases of peripheral facial nerve palsy prove to be intractable and these treatments result in unsatisfactory outcomes in about 10% of all patients with severe Bell’s palsy and 30% of all patients with severe Ramsay Hunt syndrome [5, 9–11]. These patients experience persistent facial contracture and synkinesis which can present both physical and social challenges.

Such unresponsive patients could potentially benefit from new regenerative medical approaches to the treatment of facial nerve palsy which are a focus of much basic research. However, regenerative medicine is still in its infancy and researchers are working to determine the most effective substances for use. Among the candidate substances for such regenerative medical treatments are basic fibroblast growth factor (bFGF) [12], hepatocyte growth factor (HGF) [13], nerve growth factor (NGF) [14], brain-derived neurotrophic factor (BDNF) [15], and neuregulin 1 [16]. These substances have been reported to ameliorate facial nerve palsy in animal models with neural damage outside of the temporal bone such as caused by trauma or a tumor. However, the reported effects were partial, and considerable research is still needed to achieve clinical application for any of these substances.

One additional candidate which is promising and has attracted considerable attention among researchers is insulin-like growth factor 1 (IGF-1). IGF-1 is a small peptide (7.5 kDa) naturally produced mainly in the liver, with lower levels within the brain and peripheral nerves which is significant for facial nerve palsy [17, 18]. IGF-1 acts in many ways on the nervous system by promoting the development and growth of neurons and glial cells; differentiation of Schwann cells and their migration to axons; and neurite outgrowth and neuronal survival [19, 20]. In addition, IGF-1 has been shown to increase in studies on the sciatic nerve when peripheral nerves are injured and to promote the regeneration of nerves by stimulating the sprouting of axons leading to overall regeneration of nerves and functional recovery [21–24].

In order to study the efficacy of IGF-1 in the treatment of peripheral facial nerve palsy, we needed to determine: (1) a reliable and reproducible animal model to simulate peripheral facial nerve palsy for the purposes of preclinical testing; (2) where and how should IGF-1 be administered to achieve a good outcome; (3) what evaluation methods should be used and can they be improved; and (4) whether IGF-1 receptors are actually present for IGF-1 to act upon. We ultimately designed a guinea pig animal model of peripheral facial nerve damage which is built largely on already reported animal models [25], but was modified to better reflect the clinical conditions associated with peripheral facial nerve palsy. This improved animal model allowed us to then test the efficacy of IGF-1 on a compressed facial nerve when IGF-1 is topically administered in a gelatin-based sustained-release hydrogel (MedGel; MedGel Corporation, Tokyo, Japan) via an intratemporal route. The efficacy of IGF-1 on the compressed facial nerve was evaluated based on the degree of eyelid closure using an improved video-based measurement system; post-damage recovery rate; electroneurography (ENoG) to obtain the amplitude of the compound muscle action potential (CMAP) for the facial nerve via the nasal muscle; and the number of axons on the facial nerve. Finally, we further conducted a separate study to look for the post-nerve-damage presence of IGF-1 receptors.

We were able, using this improved peripheral facial nerve palsy animal model, to test the efficacy of IGF-1 as a regenerative agent in the treatment of peripheral facial nerve palsy. Our results showed, as described herein, that IGF-1 is a promising substance and should be further studied as a treatment option for peripheral facial nerve palsy patients diagnosed with Bell’s palsy or Ramsay Hunt syndrome.

## Materials and methods

### Reagents

IGF-1 (Mecasermin; Somazon) was purchased from OrphanPacific Pharma (Tokyo, Japan).

### Animals

Four-week-old male Hartley guinea pigs (*Cavia porcellus*, Kumagai-shigeyasu Co., Ltd. Sendai, Japan) weighing 250 to 350 g were used in this study. All guinea pigs were maintained in the same environmental conditions with free access to water and food until the end of the experiment. All procedures were approved by the Ethical Review Committee of Yamagata University Faculty of Medicine (Project Identification No. 28018) and the Institutional Review Board in accordance with the Guiding Principles for the Care and Use of Animals in the Field of Physiological Sciences and followed the Guidelines for Animal Experimentation of Yamagata University Faculty of Medicine (Approval No. 28.14.210.137).

### Preparation of the animal models

#### IGF-1 administration study

**Fig. 1:**
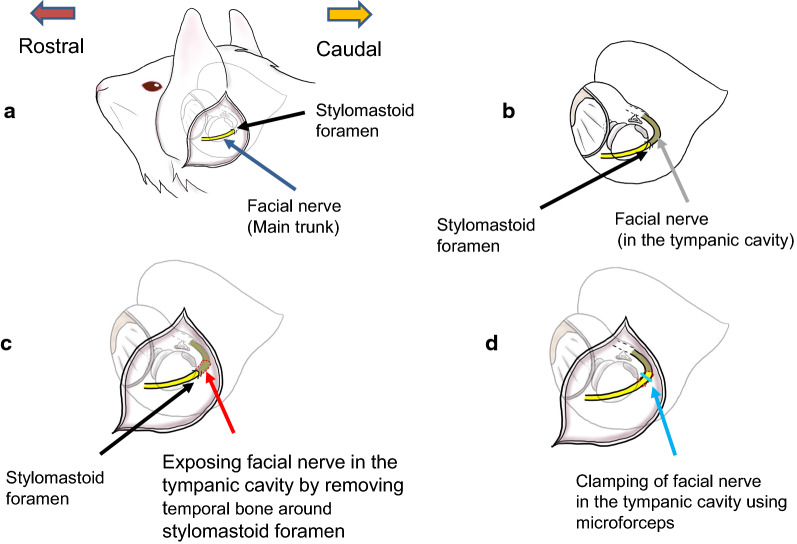
Preparing the guinea pig model of facial nerve palsy. **a** Exposing the facial nerve and the stylomastoid foramen. **b** Running of the facial nerve through the tympanic cavity. **c** Exposing facial nerve in the tympanic cavity by removing temporal bone around stylomastoid foramen. **d** Creating a compression injury by clamping the facial nerve using microforceps in the tympanic cavity

Twelve guinea pigs were divided into two groups: a saline-treated control group and an IGF-1-treated group. The guinea pigs were anesthetized before surgery with subcutaneous medetomidine (0.3 mg/kg; Kyoritsu Seiyaku Corporation, Tokyo, Japan), midazolam (4.0 mg/kg; Astellas Pharma, Tokyo, Japan) and butorphanol (5.0 mg/kg; Meiji Seika Pharma Co., Tokyo, Japan). Surgery was performed only on the left side with the right side remaining untouched as shown in Fig. 1. Temporal bone was removed under an operating microscope from around the stylomastoid foramen of the ear using drills and bone curettes to expose the tympanic cavity (Fig. 1c). The vertical portion of the facial nerve was carefully exposed from the stylomastoid foramen to 3 mm proximally and a compression injury was created in this exposed portion by clamping the nerve for 10 min using microforceps (BM563R, Castroviejo, Aesculap, B Braun, Melsungen, Germany) with a rachet lock handle. This locked handle eliminated variations in pressure which would arise if pressure was exerted by hand and further ensured that the pressure and damage to the nerve was consistent among all animals and between groups. The compression pressure of the microforceps was measured prior to surgery and found to be 35 MPa using a pressure measurement film (Prescale, MS, FUJIFILM Corporation, Tokyo, Japan).

The test substances were administered based largely on the protocol of Iwai et al. [26] as described below. Both groups had the test substances administered topically onto the exposed nerve via an intratemporal route. The saline-treated group (*n* = 6) was administered 4 mg of gelatin-based sustained-release hydrogel (MedGel; MedGel Corporation, Tokyo, Japan) saturated with 40 µL saline while the IGF-1-treated group (*n* = 6) was administered 4 mg of the same gelatin-based sustained-release hydrogel saturated with 40 µL saline containing 400 µg of IGF-1 (Mecasermin; Somazon, OrphanPacific Pharma, Tokyo, Japan). The wound was then sutured closed and the animal was placed on a heating pad. Once the anesthesia had worn off, successful nerve compression was confirmed by the lack of eyelid closure or whisker movement response to an external stimulus.

#### IGF-1 receptor study

Fifteen guinea pigs were anesthetized before surgery with subcutaneous medetomidine (0.3 mg/kg; Kyoritsu Seiyaku Corporation), midazolam (4.0 mg/kg; Astellas Pharma) and butorphanol (5.0 mg/kg; Meiji Seika Pharma Co). All animals underwent the same surgical procedure as described for the above IGF-1 administration study up to and including the exposure of the vertical portion of the facial nerve. At this point, 8–10 mg of the normal facial nerve was harvested from 3 mm distal to the stylomastoid foramen from three animals. The remaining 12 animals received a compression injury, again as described above, and 8–10 mg of the compressed facial nerve was harvested from 3 mm distal to the stylomastoid foramen in three animals each at 1 day, 2 days, 7 days, and 14 days after surgery.

### IGF-1 administration study data collection

#### Evaluation of eyelid closure

**Fig. 2:**
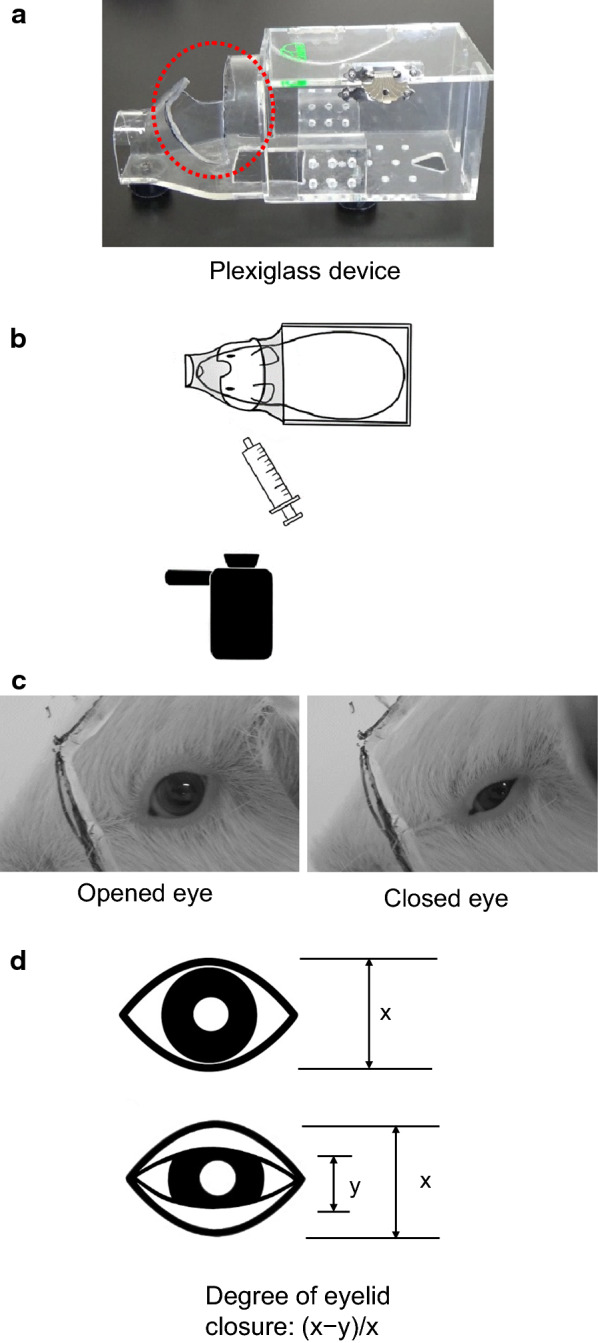
Immobilization of the guinea pig and the images of opened eye and closed eye captured from video recording data. **a** Plexiglass device. **b** Immobilization of guinea pigs and blowing air with a syringe. **c** Image of an opened eye and a closed eye. **d** The degree of eyelid closure defined as “(*x* − *y*)/*x*”. A greater degree of eyelid closure suggests a better recovery from facial nerve palsy

Eyelid closure ability was examined using an air stimulus with the response recorded with a video camera (HDR-CX535, Sony, Tokyo, Japan) set at the 60 fps mode at 2, 3, 4, 5, 6, 7, and 8 weeks postoperatively to evaluate the functional recovery of the facial nerve. Eyelid closure was examined in the left eye of both the saline-treated control group and the IGF-1 treated group by blowing a jet of air onto the eye. The animal was immobilized in a plexiglass device with a hole opened to allow access to the eye as shown in Fig. 2a, b. The camera was set up 30 cm from the front edge of the immobilization device, held by hand and steadied with the elbow after which fine adjustments were made to ensure that the left eye of the animal was located in the center of the picture frame. Eyelid closure was first evoked by blowing a 10-mL jet of air with a 20-mL syringe onto the left eye at a distance of 3 cm, which was defined as a strong stimulus, and then approximately 10 s later at a distance of 6 cm, which was defined as a weak stimulus. The 3-cm and 6-cm distances were also measured from the front edge of the immobilization device. The jet pressure was measured as 0.65 kPa at the tip of the syringe, and air speed was measured 0.28 m/s at 3 cm and 0.19 m/s at 6 cm using an anemometer (Anemomaster 6115; Kanomax, Osaka, Japan).

Two frames were selected from the recorded video data at both distances of 3 cm and 6 cm from each trial for each animal. Specifically, the frame showing the eye opened to the widest degree and the frame showing the eye closed to the narrowest degree. Precise measurements were calculated for the degree of eyelid closure for each of the selected frames using ImageJ, an open source image processing program [27] (Fig. 2c).

#### Calculation of degree of eyelid closure and recovery rate

The degree of eyelid closure was calculated as shown in Fig. 2d. In the present study, we defined complete recovery as 100% eyelid closure and incomplete recovery as less than 100% eyelid closure at 8 weeks postoperatively. In advance of this research, we had confirmed that the degree of eyelid closure was 100% in untreated guinea pigs.

#### Electrophysiological evaluation by ENoG for collection of CMAP data

**Fig. 3:**
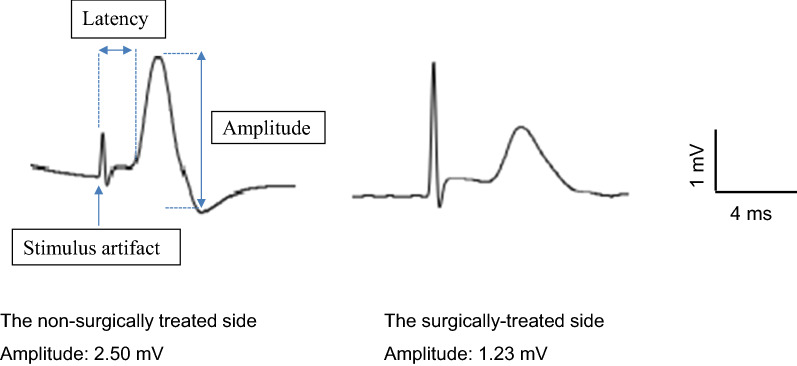
Electroneurography (ENoG). Amplitude, latency and stimulus artifact are as defined in the figure. In this case, the amplitude of the non-surgically treated side is 2.50 mV and the amplitude of the surgically treated side is 1.23 mV. The decrease in the CMAP amplitude in the surgically treated side is caused by the damage to the axon and demyelination, while the increase in latency is caused by demyelination

The CMAP amplitude reflects the number of conducting axons and the conduction velocity. Thus, the decrease in the CMAP amplitude in the surgically treated side is caused by the damage to the axons and a delay in the conduction velocity. The CMAP amplitude was used to evaluate the electrophysiological response of both groups. The CMAP data were collected under anesthesia administered using the same anesthesia protocol as during the initial surgery. The CMAP of the left facial nerve were recorded via the nasal muscle for each animal in both groups at 8 weeks postoperatively using electromyography (Power Lab 26T, Bio Research, Nagoya, Japan). The monopolar needle electrodes were inserted into the nasal muscle, and a ground electrode was positioned on the latissimus dorsi muscle. Facial nerves were supramaximally stimulated with a rectangular current pulse of 0.1 ms in duration at the stylomastoid foramen, and electromyograms of nasal muscles were recorded and a sampling rate of 40 kHz with an amplification range of ± 200 μV to ± 20 mV. Figure 3 shows the CMAP recordings for the nasal muscle on the non-surgically treated side (right) and the surgically treated side (left). The first biphasic waveform is a stimulus artifact, which is ignored, and the second biphasic waveform is the CMAP. The decrease in the CMAP amplitude on the surgically treated side is caused by the damage to the axon and demyelination while the increase in latency is caused by demyelination. We perform a statistical analysis on both groups to verify whether the CMAP amplitude correlates with the degree of eyelid closure at 8 weeks postoperatively. It should be noted that the instrument which we used (Power Lab 26T) does not allow for measurement of the electrode resistance and furthermore we did not use a digital filter.

#### Determination of number of axons in facial nerve

After ENoG testing at 8 weeks postoperatively, all animals were sacrificed under anesthesia and perfused transcardially with phosphate buffered saline (PBS), followed by 4% paraformaldehyde in PBS. Then, a 5-mm length of the facial nerve at the extratemporal portion was harvested from 3 mm distal to the stylomastoid foramen and embedded in OCT Compound (Funakoshi, Tokyo, Japan) at 20 °C. The frozen specimens of the facial nerve were cut in 8 µm sections using a cryostat (HM525 NX, Thermo Fisher Scientific, Waltham, MA, USA) and stored at − 20 °C. The axons were then immunofluorescence stained. The samples were rinsed with PBS and incubated in blocking solution (5% bovine serum albumin (BSA) with 0.3% Triton X-100 and 20% Block Ace) for 30 min at room temperature (RT). The specimens were then incubated with the following primary antibodies: rabbit polyclonal anti-neurofilament 200 (1:1000; Sigma Aldrich, Cat. No. N4142, RRID: AB_477272, St. Louis, MO, USA) in 1% BSA with 0.3% TritonX-100 and 20% Block Ace in PBS overnight at 4 °C. Subsequently, they were incubated with Alexa Fluor 568 conjugated anti-rabbit donkey IgG (1:500; Thermo Fisher Scientific, Cat. No. A10042, RRID: AB_2534017) as secondary antibodies in PBS for 2 h at RT. The number of axons in each specimen was counted using a fluorescence microscope with an automatic counting system (BZ-700, Keyence, Osaka, Japan) under search conditions where axons are defined as having a diameter measuring from 2 to 10 μm. This inclusion criterion was set down based on the protocols of Matsumoto [28] which reported that signals outside this range were not likely to be nerves.

### IGF-1 receptor study data collection

#### Measurement of IGF-1 receptor mRNA by quantitative reverse transcription PCR (qRT-PCR)

The expression of IGF-1 receptor mRNA was investigated within the facial nerve over time by qRT-PCR to confirm whether an IGF-1 receptor is present in the facial nerve. Total RNA was extracted from the 8–10 mg of the nerve harvested distal to the stylomastoid foramen using Trizol Reagent (Invitrogen, Carlsbad, CA, USA). cDNA was synthesized from 0.3 to 1.0 µg of total RNA by using a PrimeScript first-strand cDNA synthesis kit (Takara, Shiga, Japan). qRT-PCR analysis was then performed with PowerUp SYBR Green Master Mix (Thermo Fisher Scientific) in a StepOnePlus System (Thermo Fisher Scientific, RRID: SCR_015805).

The relative standard curve method was used to determine the relative mRNA expression, using β-actin as the reference. The specific primers used in this study were designed using a Basic Local Alignment Search Tool (http://blast.ncbi.nlm.nih.gov/Blast.cgi, RRID: SCR_004870) as follows: IGF1-R: forward, 5′-TCCAAGGGTGCACCATCTTC-3′, and reverse, 5′-AGACCAAGGCATGGGAATGG-3′, β-actin: forward, 5′-ATTGCCGACAGGATGCAGAA-3′, and reverse, 5′-CTGCTGGAAGGTGGAGAGTG-3′. All primers used in the present study were assessed for analytical sensitivity and specificity. PCR was performed in a 20 μL mixture containing 10 μL of 2 × PowerUp SYBR Green Master Mix Premix, 2 μL (500 nM) of each primer, 2 μL of 2.5 × IGF-1 DNA template or 10 × β-actin DNA template, and 6 μL of distilled H_2_O. The PCR cycle conditions consisted of uracil-DNA glycosylase activation at 50 °C for 2 min and DNA polymerase activation at 95 °C for 2 min, followed by 40 cycles of denaturation at 95 °C for 15 s and annealing/extension at 60 °C for 60 s.

### Statistical analysis

Results are indicated as the mean ± SE or median (interquartile range [IQR]) as appropriate. The Mann–Whitney *U* test was used to compare the degree of eyelid closure, CMAP amplitudes, and numbers of axons between the saline-treated group and IGF-1-treated group. The Fisher’s exact test was used to compare the complete recovery rates between the two groups. To get a better picture of the outcome achieved over time as opposed to simply comparing single points in time, we used the repeated measures linear mixed model containing terms for treatment (IGF-1 or saline), time period (0 weeks, 2 weeks, 3 weeks, 4 weeks, 5 weeks, 6 weeks, 7 weeks, and 8 weeks) and treatment- × -time interaction with an unstructured covariance structure [29, 30]. A Spearman’s rank correlation coefficient was calculated to measure the correlation between the CMAP amplitude and the degree of eyelid closure. A multi-comparison test procedure was performed using the Kruskal–Wallis test followed by the Steel–Dwass test to compare the IGF1 receptor expression levels over time. All statistical analyses were performed using Stat Mate version 4 software (ATMS, Tokyo, Japan) and STATA 16.0 (StataCorp), and *P* < 0.05 was considered significant in the tests of statistical inference.

## Results

### Evaluation of eyelid closure

**Fig. 4:**
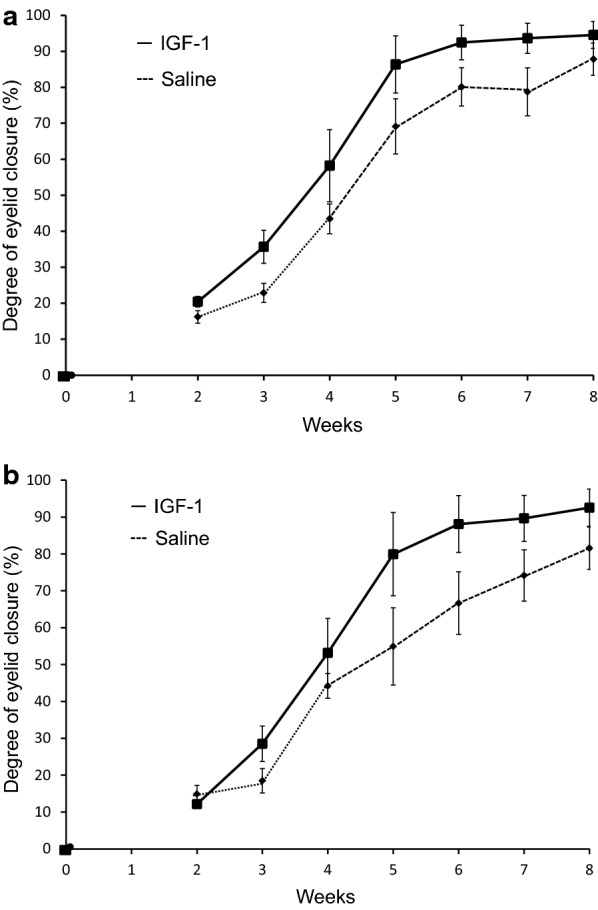
Changes in the degree of eyelid closure over time. **a** Changes in the degree of eyelid closure evoked by an air jet from a distance of 3 cm. **b** Changes in the degree of eyelid closure evoked by an air jet from a distance of 6 cm. Values represent the mean ± SE. *n* = 6 (*n* = 4 in the saline-treated group for 2 and 3 weeks postoperatively)

The changes in the degree of eyelid closure evoked by an air jet from a distance of 3 cm and 6 cm are shown in Fig. 4a, b. First, we investigated the degree of eyelid closure at each postoperative time period (i.e., 2 weeks, 3 weeks, 4 weeks, 5 weeks, 6 weeks, 7 weeks, and 8 weeks) at both air jet distances. The IGF-1-treated guinea pigs exhibited a higher degree of eyelid closure compared to the saline-treated group, but the difference was not statistically significant at either distance at all individual measurement points. The final measurements taken at 8 weeks postoperatively revealed higher values for the IGF-1-treated group (3 cm: 94.5 ± 3.7%, 6 cm: 92.6 ± 5.1%) versus the saline-treated group (3 cm: 87.8 ± 4.5%, 6 cm: 81.6 ± 5.7%), but again the differences were not statistically significant (*P* = 0.132).

However, different animals can exhibit different healing rates that can hide the ultimate improvement over time when only single points in time are compared. Thus, we also employed the repeated measures linear mixed model which is now widely used in medical research because it allows a comparison of recovery over the total time period rather than requiring only a comparison at a single point in time. In this model, the values for the saline group are set to zero for changes in the degree of eyelid closure from 2 to 8 weeks postoperatively and compared to the IGF-1 group using the terms for treatment, time, and treatment-by-time interaction with an unstructured covariance structure (*P* = 0.027). The difference in the changes of the degree of eyelid closure elicited by the air jet at a distance of 6 cm from 2 to 8 weeks postoperatively between IGF-1-treated and saline-treated guinea pigs was significant. This finding demonstrated that the degree of eyelid closure evoked by the air jet at a distance of 6 cm in the IGF-1-treated guinea pigs significantly increased from 2 to 8 weeks postoperatively. The difference in the changes in the degree of eyelid closure evoked by the air jet at a distance of 3 cm from 2 to 8 weeks postoperatively between the two groups was not significantly different in the repeated measures linear mixed model (*P* = 0.446).

### Recovery rate

Complete recovery of 100% degree of eyelid closure was observed at 8 weeks postoperatively in four out of the six guinea pigs in the IGF-1-treated group while no animals exhibited complete recovery in the saline-treated group. The complete recovery rate in the IGF-1-treated group at 66.7% was significantly greater than that in the saline-treated group at 0% (Table 1) (*P* = 0.014).

**Table 1:** The rates of complete recovery at 8 weeks postoperatively

	Complete	Incomplete	Complete recovery rate (%)
3 cm			
Saline	0	6	0
IGF-1	4	2	67
6 cm			
Saline	0	6	0
IGF-1	4	2	67

The complete recovery rates in the IGF-1-treated guinea pigs were greater than in those in the saline-treated group. *n* = 6. *P* = 0.014

Moreover, the animals in the IGF-1-treated group could be divided in a responder group of 4 animals vs. a non-responder group of 2 animals. The results obtained for the non-responder group were the same or worse than those results obtained in the saline-treated group.

### Electrophysiological evaluation

**Fig. 5:**
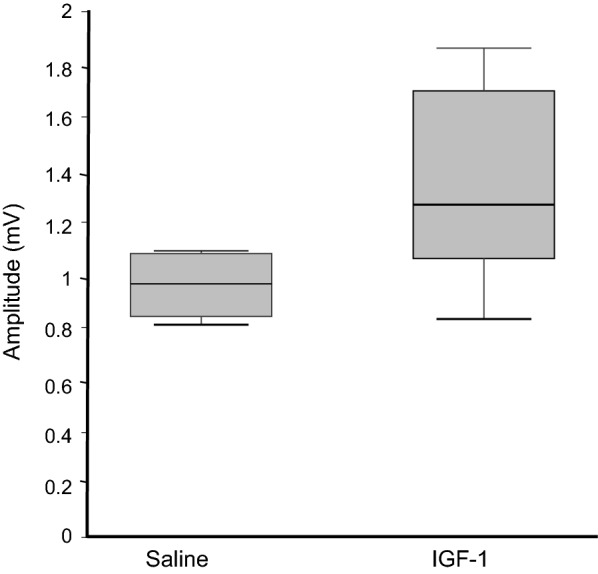
The CMAP amplitude in the IGF-1-treated and saline-treated groups. No significant difference was observed between the two groups (*P* = 0.094), *n* = 6

Figure 5 shows the CMAP amplitude for each group. No significant difference was found in the median amplitude between the IGF-1-treated group at 1.27 (1.06–1.7) mV versus the saline-treated group at 0.97 (0.84–1.08) mV (*P *= 0.094). In addition, no significant difference was found in the median latency between the IGF-1-treated group at 2.23 (2.00–2.82) ms versus the saline group at 2.40 (2.00–2.62) ms (*P* = 0.818) (data not shown).

### Correlation of the CMAP amplitude and the degree of eyelid closure

**Fig. 6:**
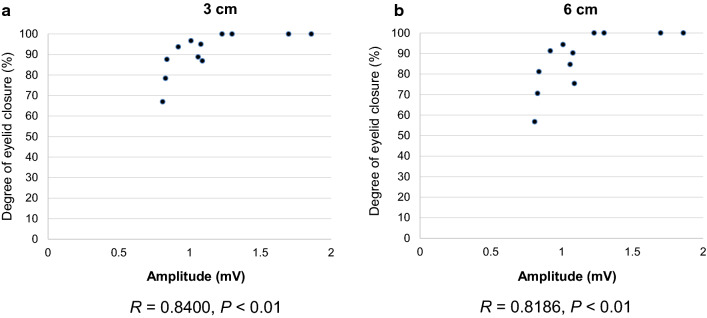
Correlation of the CMAP amplitude and the degree of eyelid closure. **a** Correlation of the CMAP amplitude and the degree of eyelid closure evoked by an air jet from a distance of 3 cm. **b** Correlation of the CMAP amplitude and the degree of eyelid closure evoked by an air jet from a distance of 6 cm. The CMAP amplitude exhibited a positive monotonic correlation with the degree of eyelid closure at 8 weeks postoperatively (3 cm: *R* = 0.840, *P* < 0.05; 6 cm: *R* = 0.819, *P* < 0.05), *n* = 12

Figure 6 shows the correlation of the CMAP amplitude and the degree of eyelid closure evoked by an air jet from a distance of 3 cm and 6 cm. The CMAP amplitude exhibited a positive monotonic correlation with the degree of eyelid closure at 8 weeks postoperatively (3 cm: *R* = 0.840, *P* < 0.05; 6 cm: *R* = 0.819, *P *< 0.05).

### Number of axons

**Fig. 7:**
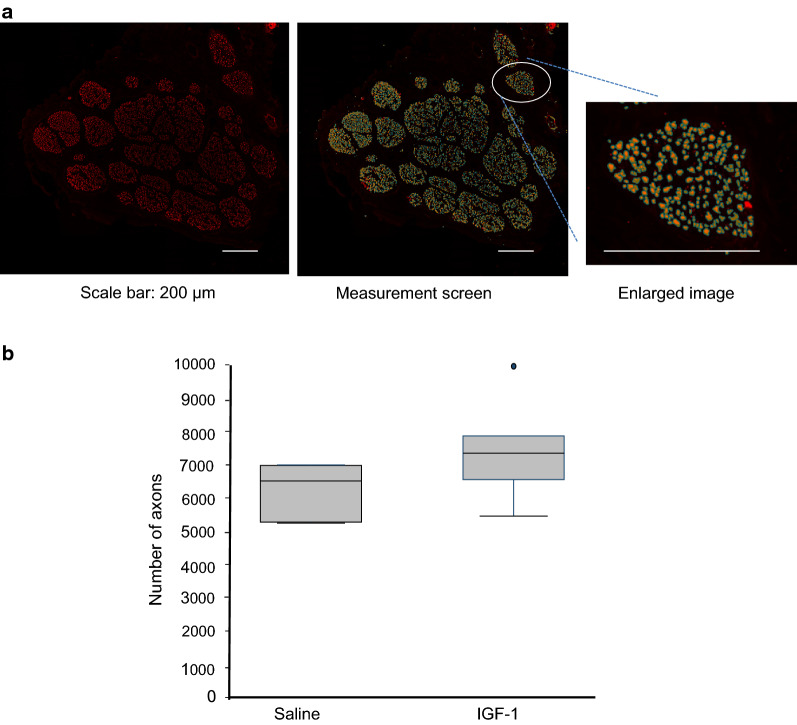
Histopathology of the facial nerve by immunofluorescence staining using anti-neurofilament 200. **a** The axons counted with an automatic counting system are seen as yellow in an enlarged image. Scale bar: 200 µm. **b** The number of axons at 8 weeks postoperatively. No significant difference was observed between the IGF-1-treated group and the saline-treated group. Saline, *n* = 6; IGF-1, *n* = 5. *P* = 0.484

The number of axons was counted as shown in Fig. 7a with the number of axons were in the IGF-1-treated group (*n* = 5) at 7342 (6542–7861) versus in the saline-treated group (*n* = 6) at 6509 (5269–6972) (Fig. 7b). However, the difference was not found to be significant (*P* = 0.484).

### IGF-1 receptor mRNA expression

**Fig. 8:**
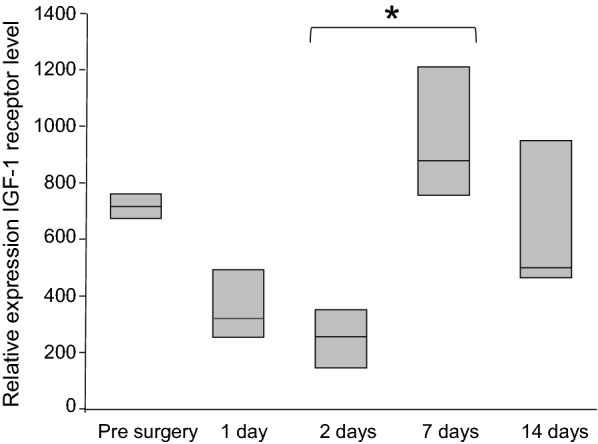
Changes in the expression of the IGF-1 receptor mRNA within the facial nerve. The relative quantification of the IGF-1 receptor mRNA was significantly greater at 7 days after compression than at 2 days after compression. *n* = 3. *P = 0.031

The IGF-1 receptor mRNA was found to be expressed in normal facial nerves and in the compressed nerves at all measurement points (Fig. 8). The median of the relative quantification of the IGF-1 receptor mRNA was 717 (673–761) in the normal facial nerves; 321 (253–492) at 1 day after compression; 255 (144–350) at 2 days after compression; 877 (755–1210) at 7 days after compression; and 500 (464–949) at 14 days after compression. When comparing IGF-1 receptor mRNA expression over time by using the multi-comparison test, the relative quantification of the IGF-1 receptor mRNA was significantly greater at 7 days after compression than at 2 days after compression (*P* = 0.031).

## Discussion

Our objective herein is to demonstrate that IGF-1 shows promise as a regenerative medical treatment protocol for intractable peripheral facial nerve palsy caused by Bell’s palsy or Ramsay Hunt syndrome and that IGF-1 receptors are present in the facial nerve. We first confronted the question whether an existing animal model was sufficient or whether we needed to develop a new model to better simulate clinical conditions for peripheral facial nerve palsy. We further considered the best method by which IGF-1 could be administered. When conducting a preclinical study of the use of a regenerative agent such as IGF-1 on a condition such as peripheral facial nerve palsy, the model would obviously focus on the facial nerve. The facial nerve runs from the pons, passes through the temporal bone via the facial canal and travels out of the cranium at the stylomastoid foramen [31]. A number of studies have employed facial nerve damage models in order to examine the effects of different therapies, but these studies focus on clamping or cutting a distal portion of the facial nerve outside of the temporal bone because it is easy to expose the facial nerve running subcutaneously outside of the temporal bone [32–34]. These models generally do not accurately reflect the situation experienced by patients with peripheral facial nerve palsy.

One reason that these models have avoided the portion of the facial nerve within the temporal bone is that the space available for accessing the facial nerve within the temporal bone is narrow and considerable surgical skill is required to manipulate the intratemporal facial nerve. Komobuchi et al. developed an animal model which focuses on the facial nerve within the temporal bone and better simulates the clinical pathology of Bell’s palsy or Ramsay Hunt syndrome [25]. They opted to surgically expose the facial nerve from the vertical portion all the way to the horizontal portion and then subsequently temporarily clamped only the vertical portion of the nerve to induce damage. Komobuchi’s model represents a considerable improvement over previous peripheral facial nerve palsy animal models, and we adopted this model with a few modifications. These modifications covered the surgical procedure, method of substance administration, and methods for evaluating the results.

Our primary modification was in the surgical procedure in which the objective was to limit the damage to the nerve to that damage caused by clamping. We tried to achieve this objective by only exposing the vertical portion of the facial nerve which is a relatively non-invasive procedure and requires only a dermal incision and removal of a minimal amount of bone. In contrast, exposing the horizontal portion would require removal of a considerable amount of bone and potentially other surrounding tissue and vascular structures. Thus surgical exposure and clamping of only the vertical portion of the facial nerve should not only result in the aforementioned limiting nerve damage to that caused only by clamping but also minimize non-nerve extraneous damage that could affect recovery and/or regeneration of the facial nerve. We also confirmed in a pilot study that intratemporal facial nerves could be exposed without inducing measurable facial palsy.

Furthermore two issues need to be directly addressed. The first issue is the difference in the time frame between the histological examination of the presence of IGF-1 receptors which was conducted up to 14 days versus the recovery of facial nerve function which was examined from 14 days. The second issue is the time lag between the presence of IGF-1 receptors and administration of IGF-1 versus recovery of facial nerve function. The IGF-1 receptors were only examined up to 14 days after surgery and the administration of IGF-1 which was only efficacious for 14 days, however, the recovery of facial nerve function in terms of eyelid closure and whisker movement response appeared only slightly at the 2-week mark after which substantial improvement was observed in the absence of IGF-1. Both the difference in the examination time frame and the recovery time lag can be explained by the physiology of axonal nerve recovery after a crush or cut injury which can be broadly classified as due to any or all of three types of nerve injury: trauma due to physical injury; vascular injury due to ischemia; and chemical injury due to neurotoxicity [35]. The resultant nerve injury can also be broadly classified into three types: neuropraxia, axonotmesis and neurotmesis [36] based on the degree of damage to the nerve. Neuropraxia represents minor injury which generally recovers without intervention while neurotmesis represents severe injury resulting from a severing of the nerve. Our model was designed to replicate axonotmesis which can range from degeneration to just an axon and myelin sheath up to degeneration to an axon, myelin sheath, endoneurium and perineurium but not the epineurium. The preservation of the outer epineurium creates an enclosed environment for regeneration and prevents the total transection of the nerve as seen in neurotmesis. However, a lag is observed between healing versus regeneration and restoration of function of the nerve. When a nerve fiber is crushed or cut, the part of the axon distal to the injury degenerates for 1 to 2 weeks in a process known as Wallerian degeneration. It is during this 2-week time period that IGF-1 is present and is thought to activate the signal transduction system in the cell and promote overall recovery. However, regeneration of axon fibers only takes place after the degeneration process has ended which explains both the delay in recovery of nerve function in all animals and the superior results observed in the IGF-1 treated animals.

We further strove to improve our animal model in two additional ways: first by a topical administration of IGF-1 and by a sustained-release administration method. Nakagawa et al. have previously demonstrated the superiority of the topical application of IGF-1 to the round window niche using a sustained-release hydrogel in patients with sudden deafness who do not respond to systemic administration of corticosteroids [37]. Tabata et al. further investigated the sustained-release properties in vivo of the same type of gelatin-based hydrogel which we employed but containing bFGF instead of IGF-1 and they reported that the release of bFGF persisted over 2 weeks [38]. In line with these findings, we topically administering IGF-1 intratemporally onto the damaged nerve using the sustained-release, gelatin-based hydrogel MedGel as previously described above. This method of administration, if used in a future clinical setting, would allow IGF-1 to be administered a single time to the damaged nerve via minimally invasive transcanal endoscopic ear surgery (TEES) [39–41].

Another area which we strove to improve upon was the collection and evaluation of the appropriate quantitative data to determine the efficacy of IGF-1 on the test animals. Several methods have been reported to evaluate facial movements, such as eyelid closure and/or whisker motor performance which are, by their nature, difficult to elicit and measure in laboratory animals [7, 42, 43]. Furthermore, quantitative measurement has been a challenge because these methods often depended on the naked eye or video recording methods that were not described in detail and may have been measured by hand. We found that degree of eyelid closure could be accurately measured and evaluated by video recording and computer analysis as described above, but that whisker motor performance was not amenable to accurate analysis due to the number and size of the whiskers along with their speed and angle of movement. Thus, to more accurately record and evaluate post-treatment recovery of the test animal, we focused on the eyelid movement as expressed by degree of eyelid closure; degree of recovery; and the CMAP amplitude. Our findings as shown in Fig. 6 demonstrated a positive monotonic correlation between the degree of eyelid closure and CMAP amplitude which points to the usefulness of this method.

The most noteworthy finding was that of the complete recovery rate of 66.7% for the IGF-1-treated group in comparison to 0% for the saline-treated group. If this 66.7% complete recovery rate could be even partially duplicated in a clinical trial, it would represent a huge improvement over the current situation in the treatment in intractable peripheral facial nerve palsy. What is also striking is that those animals in the IGF-1-treated group which did not achieve a complete recovery exhibited values that were the same or worse than those observed in the saline-treated group. This finding suggests that a physiological basis may be behind the response or non-response of an animal to IGF-1 treatment. What this physiological basis could be is well beyond the scope of this paper, but this dramatic difference in intragroup response should not be ignored and needs to be further examined.

One physiologically aspect of IGF-1 which was examined here and needs to be examined in much greater depth in the future is the presence of IGF-1 receptors. We assumed that IGF-1 restored facial nerve function by activating signaling downstream of the IGF-1 receptor. Many experiments have been conducted in vitro to investigate the mechanism of nerve regeneration by IGF-1 via signaling pathway downstream of the IGF-1 receptor. IGF-1 promotes the myelinating phenotype of Schwann cells in dorsal root ganglion neuron/Schwann cell cocultures, via the PI3K/Akt signaling pathway [44]. Liang et al. demonstrated that IGF-1 plays an important role during myelin membrane formation, which activates PI3K/Akt signaling pathway and results in stimulation of fatty acid synthesizing enzymes [45]. Jones et al. found that IGF-1 stimulates the PI3K/Akt pathway and promoted axonal growth [46], and Liu et al. demonstrated that IGF-1 enhances GAP-43 expression via the PI3K/Akt pathway, which results in axonal growth [47]. We have speculated that topical application of IGF-1 activates the PI3K/Akt pathway in vivo, while at the same time, the topical application of IGF-1 does not appear to contribute to recovery of the myelin sheath.

Our review of the literature did not uncover any studies of the presence of IGF-1 receptors in the facial nerve. However, Cheng et al. reported in 1996 on the presence and expression of IGF-1 receptors after sciatic nerve transection in rats [48]. Xu et al. also confirmed the presence of IGF-1 receptors in an experiment on crush injuries of the sciatic nerve in rats [49]. We looked at the IGF-1 receptor mRNA expression within the facial nerve in guinea pigs and were also able to confirm the presence of IGF-1 receptors in the facial nerve. We found that IGF-1 receptor mRNA was expressed during the 14-day observation period after the nerve injury with the expression of the IGF-1 receptor mRNA exhibiting a small but insignificant decrease at 2-day postoperative but returning to the preoperative levels within 7 days. These findings suggest that gradual and sustained release of IGF-1 via a hydrogel should be able to take advantage of the presence of the IGF-1 receptors.

The present study has several limitations. Our guinea pig model of peripheral facial nerve palsy was produced by clamping intratemporal facial nerves, and this model is not identical to the pathology of Bell’s palsy or Ramsay Hunt syndrome, which can occur as the result of neural edema and constriction within the facial canal most likely after a herpetic viral infection. However, a facial nerve palsy animal model would be extremely difficult to create using an actual herpetic viral agent with any consistency [50, 51]. Moreover, evidence is available that suggests that peripheral facial nerve palsy classified as Bell’s palsy can have a non-viral etiology. Thus, an animal model can be considered accurate replication of the peripheral facial nerve palsy conditions if it replicates the neural edema and constriction within the facial canal as our clamping method does. Furthermore, the intracellular mechanisms of the facial nerve recovery by IGF-1 remains to be clarified in vivo. Further investigation needs to be conducted regarding the PI3K/Akt signaling pathway.

An additional limitation is that while the number of axons was the same between the IGF-1-treated group and the saline-treated group, ideally the axons need to be microscopically examined to determine whether any functional or morphological differences are present between the axons of each group. Such research would require the use of an electron microscopic which we would like to conduct as a next step in the future together with further examination of the distal portion of the axon.

Despite these limitations, our findings demonstrated the efficacy of sustained-release of IGF-1 through topical administration via an intratemporal route in a guinea pig model of peripheral facial nerve palsy. Sustained application of IGF-1 could become a new treatment option for peripheral facial nerve palsy in humans.

## Conclusion

Topical intratemporal application of IGF-1 via a sustained-release gelatin-based hydrogel resulted in an improvement in the changes of the degree of eyelid closure over the total time period as well as a significantly higher complete recovery rate for facial nerve function in an IGF-1-treated group versus a saline-treated group in a guinea pig model of peripheral facial nerve palsy simulated by a facial nerve compression injury. Additional experiments should be conducted before clinical trials for treatment of peripheral facial nerve palsy in humans.

## Data Availability

The datasets used and/or analyzed during the current study are available from the corresponding author on reasonable request.

## Abbreviations

IGF-1: Insulin-like growth factor 1
mRNA: Messenger ribonucleic acid
ENoG: Electroneurography
VZV: Varicella zoster virus
bFGF: Basic fibroblast growth factor
HGF: Hepatocyte growth factor
NGF: Nerve growth factor
BDNF: Brain-derived neurotrophic factor
CMAP: Compound muscle action potential
PBS: Phosphate buffered saline
BSA: Bovine serum albumin
RT: Room temperature
qRT-PCR: Quantitative reverse transcription polymerase chain reaction
cDNA: Complementary deoxyribonucleic acid
SE: Standard error
IQR: Interquartile range
TEES: Transcanal endoscopic ear surgery
